# Utilizing mast cells in a positive manner to overcome inflammatory and allergic diseases

**DOI:** 10.3389/fimmu.2022.937120

**Published:** 2022-09-14

**Authors:** Zhongwei Zhang, Peter B. Ernst, Hiroshi Kiyono, Yosuke Kurashima

**Affiliations:** ^1^ Department of Innovative Medicine, Graduate School of Medicine, Chiba University, Chiba, Japan; ^2^ Division of Comparative Pathology and Medicine, Department of Pathology, University of California, San Diego, San Diego, CA, United States; ^3^ Center for Veterinary Sciences and Comparative Medicine, University of California, San Diego, San Diego, CA, United States; ^4^ Department of Medicine, School of Medicine and Chiba University-University of California San Diego Center for Mucosal Immunology, Allergy and Vaccine (CU-UCSD), University of California, San Diego, San Diego, CA, United States; ^5^ Future Medicine Education and Research Organization, Chiba University, Chiba, Japan; ^6^ Division of Mucosal Immunology, IMSUT Distinguished Professor Unit, The Institute of Medical Science, The University of Tokyo, Tokyo, Japan; ^7^ Department of Human Mucosal Vaccinology, Chiba University Hospital, Chiba, Japan; ^8^ HanaVax Inc., Tokyo, Japan; ^9^ Mucosal Immunology and Allergy Therapeutics, Institute for Global Prominent Research, Chiba University, Chiba, Japan; ^10^ Research Institute of Disaster Medicine, Chiba University, Chiba, Japan; ^11^ Institute for Advanced Academic Research, Chiba University, Chiba, Japan; ^12^ Empowering Next Generation Allergist/immunologist toward Global Excellence Task Force toward 2030 (ENGAGE)-Task Force, Tokyo, Japan

**Keywords:** mast cells, heterogeneity, inflammation, allergy, tolerance

## Abstract

Mast cells (MCs) are immune cells widely distributed in the body, accompanied by diverse phenotypes and functions. Committed mast cell precursors (MCPs) leave the bone marrow and enter the blood circulation, homing to peripheral sites under the control of various molecules from different microenvironments, where they eventually differentiate and mature. Partly attributable to the unique maturation mechanism, MCs display high functional heterogeneity and potentially plastic phenotypes. High plasticity also means that MCs can exhibit different subtypes to cope with different microenvironments, which we call “the peripheral immune education system”. Under the peripheral immune education system, MCs showed a new character from previous cognition in some cases, namely regulation of allergy and inflammation. In this review, we focus on the mucosal tissues, such as the gastrointestinal tract, to gain insights into the mechanism underlying the migration of MCs to the gut or other organs and their heterogeneity, which is driven by different microenvironments. In particular, the immunosuppressive properties of MCs let us consider that positively utilizing MCs may be a new way to overcome inflammatory and allergic disorders.

## Introduction

MCs predate the emergence of acquired immunity, since they were first named in 1878, a broad understanding of MCs biology has been published (1). Recently, an investigation of the hematopoietic origin of connective tissue MCs using fate-mapping systems has revealed the dual developmental origin of MCs (2). It was found that most connective tissue MCs (CTMCs) are derived from late erythro-myeloid progenitors (EMPs) produced in the yolk sac at E8.5 (the second transient definitive wave of fetal hematopoiesis), while mucosal MCs (MMCs) seem to come from hematopoietic stem cells (HSCs) in the bone marrow in mice (3). Committed progenitor cells enter the bloodstream, where they are found as lineage-negative Lin^−^ c-Kit (CD117)^hi^ FcϵRI^+^ β7-integrin^hi^ ST2^+^ CD16/32^hi^ or FcϵRI^−^ cells (4). In human beings, Lin^−^ c-Kit^int/hi^ FcϵRI^+^ β7-integrin^+^ CD34^hi^ blood cells are considered the immediate precursor of MCs (5). These MCPs travel in the blood circulation and eventually arrive at the peripheral tissues [e.g., skin, respiratory tract, urogenital tract, gastrointestinal tract, etc. (1)] under the stimulation and induction of various factors such as chemokine receptors and adhesion molecules. For example, in the context of microbiota-influence, MCs migrate to the intestinal tract and mature under the regulation of various pathways [e.g., α4β7-MAdCAM-1/VCAM-1 (6, 7), CXC chemokine receptor 2 (CXCR2) (7) sphingosine 1-phosphate (S1P) (8)].

In the process of migration and maturation, MCs of different origins showed distinct preferences in tissue localization (6). For instance, only the adipose and pleural cavity MCs were derived from early EMPs, most of which were replaced by late EMPs during adulthood in mice (3). Late EMPs generate most of the MCs that home to connective tissues, while mucosal MCs mainly derive from fetal hematopoietic stem cells HSCs (3). Diverse tissue preferences also shape remarkable phenotypical and functional heterogeneity, suggesting that MCs may perform various functions in response to different physiological and pathological states (9).

Besides the developmental origin, differences in the tissue microenvironment are also important causes of MC heterogeneity (10). It is clear that MCs in different organs have noticeable differences (11). However, even in the same organ, the microenvironment under different pathological or physiological conditions can produce utterly distinct MC subtypes. Take some examples, in the lung, only MCs of the proximal lung express MRGPRX2, but not of the distal or medial lung (12); in the gut, both CTMCs and MMCs are present and can be transformed into each other in some cases, such as food allergy (13). All this evidence supports the remarkable microenvironment-dependent heterogeneity in MCs.

Different cellular phenotypes bring different functions—one of the representative features of the heterogeneity of MC is regulatory-like properties against inflammation and allergy. In some occasions, MCs have been shown to release cytokines to inhibit and terminate inflammatory and allergic responses (e.g., IL-2 (14) IL-10 (14–16), and TGF-β1 (17), etc.). In addition, MCs interact with regulatory T cells (Tregs) (14, 18–21) and alternatively activated macrophages (AAMΦs) (22) to inhibit inflammatory and allergic responses.

In addition, various MC-derived cytokines [e.g., IL-4, IL-8, IL-13, IL-22, TNF-α, TGF-β1, vascular endothelial growth factor (VEGF), nerve growth factor (NGF), fibroblast growth factor-2 (FGF-2), and platelet-derived growth factor (PDGF)], proteases (tryptase and chymase/chymotrypsin), histamines, lipid mediators [PGD2 and leukotrienes (LTs), etc.] participate in the process of wound healing [e.g., vascular permeability and immune cells recruitment (monocytes and neutrophils), epithelial proliferation and migration, granular formation and remodeling, and scar formation, etc.] (11, 23).

Collectively, MCs play a diverse role in various physiological and pathological processes due to their highly complex heterogeneity. A full understanding of the phenotypic characteristics and functional heterogeneity of MCs in specific diseases will help us create efficient individualized therapy.

## Aggregation of mast cells at intestinal mucosal sites

MCs exists in virtually all organs of vertebrates but different widely in their number, phenotype, and function (1). The high heterogeneity is partly attributed to their unique and complex maturation process. Although controversial (11), MCs are believed to have two main origins, bone marrow and yolk sac (2). After leaving the hematopoietic tissue, committed progenitor cells enter the blood, migrate, and colonize the target tissue [e.g., skin, respiratory tract, genitourinary tract, gastrointestinal tract, etc. (1)]. Notably, the exact ontogeny of mice adult MCs has been suggested in recent studies; early EMPs, late EMPs, and fetal HSCs are successively involved in the maturation of fetal MCs (2, 3). Using the inducible runt-related transcription factor1 *(Runx1-icre)* and colony stimulatory factor 1 receptor *(Csf1r-icre)* fate-mapping systems, Zhiqing Li et al. separately traced three waves of hematopoiesis (3). They confirmed that late EMP-derived MCs, as long-lived cells, gradually replace early EMPs and become the main contributors to the adult CTMC pool. Early EMP-derived MCs show a short lifespan in connective tissue, only appearing in adipose tissue and the pleural cavity. Fetal HSC-derived MCs, another possible type of short-lived cells, are mainly located in mucosal tissues and constantly renewed by the bone marrow (3). Therefore, there are at least two ways that MCs mature in mice. However, whether these pathways apply to other mammals, including humans, is not fully understood.

**Figure 1:**
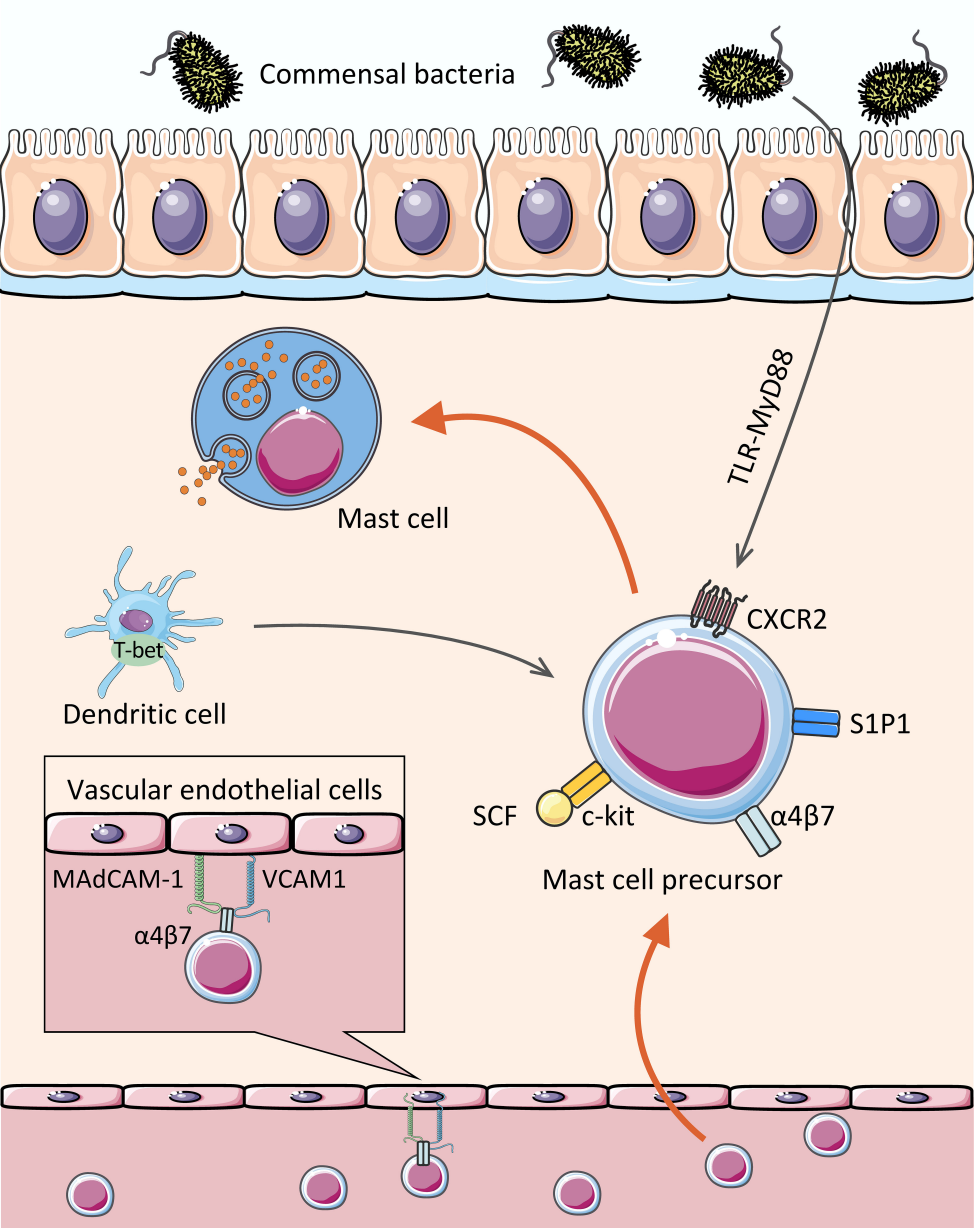
Assembly of MCs in the gut. The MCPs leave the hematopoietic tissue, enter the blood stream, migrate and colonize the target tissue. With c-kit, CXCR2, and integrin α4β7 on the cell surface, MCPs bind to MAdCAM-1 and VCAM1 on the vascular endothelial cells, thereby crossing the vascular endothelium and colonizing the intestinal mucosa and submucosa, where they mature. In addition, transcription factor (dendritic cell-derived T-bet), Lipid mediator (sphingosine 1-phosphate), and some intestinal microbiota affect the homing of MCs to the gut.

After arriving in the peripheral tissues, MCPs finally differentiate into mature MCs with the stimulation of local cytokines and growth factors in humans (e.g., IL-4 (24), IL-9 (25), IL-33 (26, 27), and TGF-β1 (28), etc.) and mice (e.g., IL-3 (29), IL-4 (28), TGF-β1 (28), and NGF (30), etc.). However, unlike other granulocytic leukocytes (e.g., neutrophils, eosinophils, and basophils), MCs mature in peripheral sites rather than bone marrow. Consequently, there are very few MCPs or mature MCs in bone marrow and blood, so it is relatively difficult to evaluate human MCs. Moreover, the molecular expression necessary for MC migration varies not only during maturation, but also according to cellular charcteristics, such CMMCs or MMCs (31, 32). All of these make it relatively difficult to study human MCs *in vitro*. So far, MCs are studied mainly in rodents (e.g., genetically modified mice) or some malignant MCs cell lines (33, 34). We herein describe some mechanisms by which MCs migrate to the intestinal tract and mature in combination with current findings (**Figure 1**).

The intestinal mucosa, which is exposed to various foreign components (including microbiota and food components), is one of the primary destinations for MCs and the pool for MCPs (35). MCs have been reported to account for approximately 2% of the cells in the lamina propria of the gastrointestinal tract (36). There is no doubt that integrin α4β7 plays a critical role in the migration of MCs to the intestinal mucosa. MCPs is utterly absent in the small intestine of α4β7-deficient mice (6). Evidence suggests that integrin α4β7 interacts with vascular endothelial molecules, including mucosal addressing protein cell adhesion molecule-1 (MAdCAM-1) and vascular cell adhesion molecule-1 (VCAM1), to facilitate directional migration of MCPs across endothelial cells to the small intestine (6, 7).

Like all blood-borne leukocytes, adhesion interactions are not the only requirement for MCPs recruitment to peripheral tissues, but also the migration to target tissues. Stem cell factor (SCF) has chemotactic effects on both mouse (37) and human (38) MCs *in vitro. In vivo*, SCF activates the c-kit expressed by MCs (or MCPs) through PI3K, then mediates migration, survival, proliferation, and activation with the help of α4 integrin (39–42). No intestinal MCPs was detected in SCF-deficient mice (6).

CXCR2 also contributes to the migration of MCs. It was experimentally demonstrated that MCs lacking CXCR2 are less able to migrate to the small intestine (7). Although the research of MC migration by CXCR2 is not detailed, the role of CXCR2 has been extensively studied in other cells. For example, in rat cardiogenic endothelial cells, the binding of CXCR2 and its ligand CXCL5 leads to downstream PI3K activation and further cytokine production (43). Another study in which human CXCR2 was stably transfected into lymphoblastic JY cell lines showed that the activation of IL-8 (CXCR2 ligand) induced transient adhesion of α4β7 dependent on VCAM-1 (44). Consequently, it is reasonable to speculate that in the circulatory system, c-kit and CXCR2 expressed by MCPs can interact with their respective ligands to upregulate the affinity of α4β7 integrin with MAdCAM-1 and VCAM-1, and also enhance the expression of VCAM-1 on epithelial cells *via* PI3K pathway (45), which further promotes the migration of MCs (7).

Previous studies have indicated that gut microbiota can mediate the constitutive migration of MCs into the intestine (46). Germ-free (GF) mice showed a lower intestinal MC density and higher blood MCs density compared to SPF (specific pathogen free) mice, followed by an impaired intestinal MCs function and maturity (reduced edema was observed after injecting the degranulation-provoking compound 48/80) (47). In detail, the expression of CXCR2 ligands, e.g., CXCL2, CXCL2, and CXCL5, were significantly reduced in the intestinal epithelial cells of GF mice and MyD88^-/-^ mice compared to the control group (46). Results revealed that the expression of CXCR2 ligands in intestinal epithelial cells induced by intestinal microbiota was at least partially dependent on the Toll-like receptor (TLR) -MyD88 pathway, which promotes MC migration to the intestine. It remains to be seen whether intestinal bacteria directly or indirectly affect MC recruitment *via* their components and metabolites.

Interestingly, intestinal migration of MCs appears to be induced by a combination of gut bacteria, rather than a single bacterial strain. A study based on the Gram-positive *Lactobacillus plantarum* provided partial evidence that a single strain did not seem to mediate MC migration (47). Martin Schwarzer et al. found GF mice showed lower levels of CXCL1 and CXCL2 in the jejunal with fewer and less mature intestinal MCs in comparison to conventional mice after the induction of food allergy. In detail, the susceptibility to allergy symptoms in GF mice was fully restored after they were cohoused with conventional mice. However, similar results were not observed in mice mono-colonized with *L. plantarum* (47). *L. plantarum* was previously shown to aggravate allergic reactions associated with degranulation of MCs (48). Thus, it remains to be determined whether other single strains (e.g., Gram-negative bacteria) or specific bacterial products affect MC migration and maturation.

In addition, T-bet, a Th1-specific T-box transcription factor expressed by leukocytes [e.g., natural killer (NK) cells, dendritic cells (DCs), and CD8^+^ T cells], seems to mediate the migration of MCPs to the intestinal tract indirectly. Pilar Alcaide et al. found that T-bet^-/-^ mice showed lower number of MCPs homing to the small intestine or lungs (49). It is worth mentioning that T-bet expressing DCs somehow contribute to the migration of MCs since MC itself does not seem to express T-bet during development (49).

Moreover, our previous studies showed that S1P mediates the migration of MCs to the large intestine  (8). S1P is a sphingolipid metabolite from platelets and MCs, which is thought to play a role in mediating the migration of lymphocytes from secondary lymphoid organs and the thymus (50). It has been know that MCs express various types of S1P receptors, including type I S1P receptor (S1P_1_) (8), associated with G_i_ protein and small GTPases (e.g., Rho and Rac) (51, 52). By administration of FTY720 to mice, a blocker of S1P_1_-mediated signaling, we found that FTY720 can directly inhibit the migration of MCs, demonstrating the requirement of S1P-S1P_1_ signaling for MC migration (8).

## Heterogeneity of mast cells and the peripheral education system according to the microenvironment

**Figure 2:**
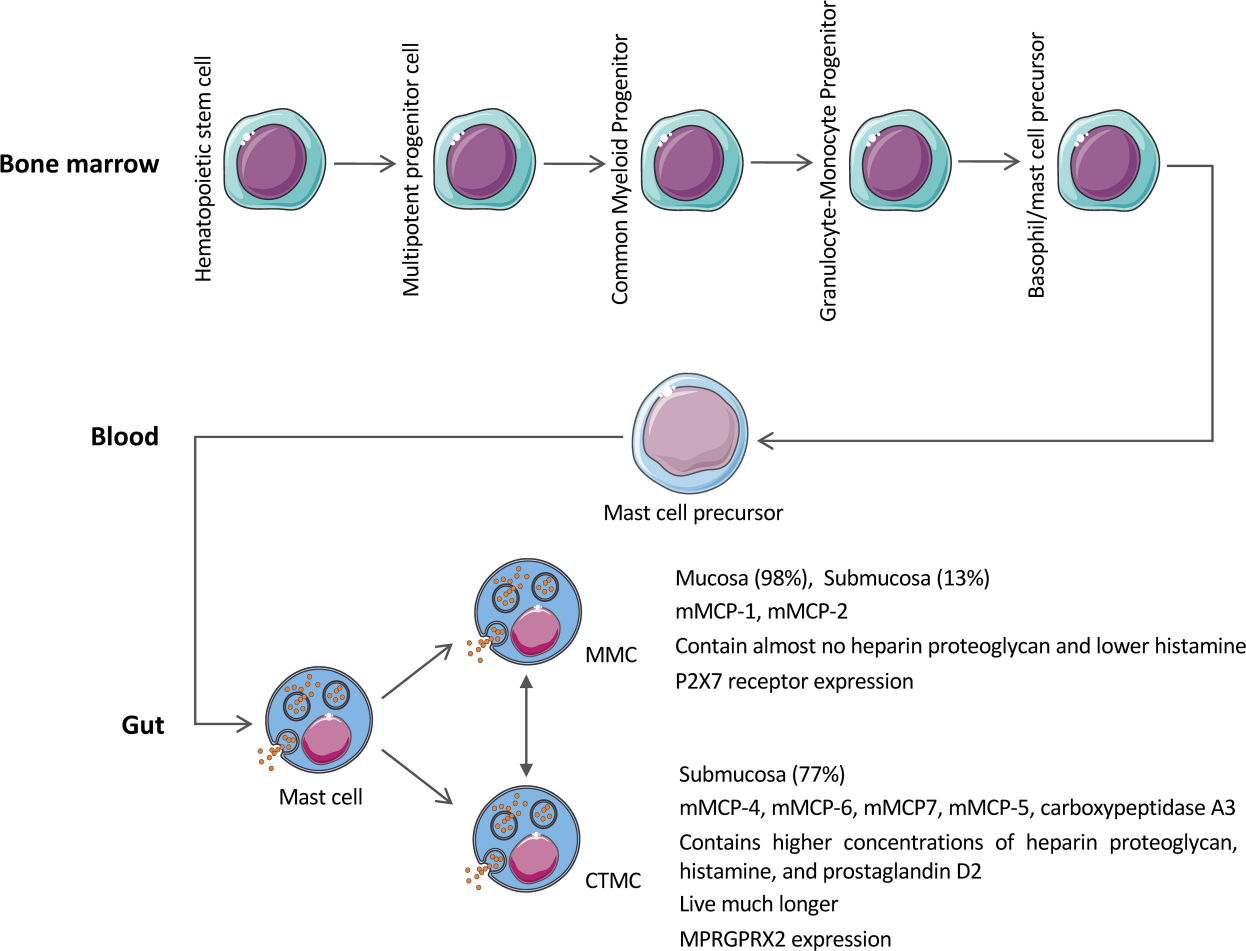
Heterogeneity of MCs. MCs mature uniquely, developing them more susceptible to the tissue homeostasis of the peripheral tissue microenvironment, and thus exhibit different subtypes and secrete different mediators to achieve different functions.

The unique maturation mechanism of MCs also contributes to their astonishing plasticity and heterogeneity, which fully reflects the complex interaction between MCs and microenvironmental signals transmitted by different tissues (10). Traditionally, MCs are classified according to the production of serine proteases, such as trypsin and chymase (11, 53). In mice, MCs are divided into MMCs and CTMCs (11, 53). MMCs, as the name implies, mainly exist in the mucosa (e.g., the intestinal mucosa) and express chymase mMCP-1 and mMCP-2. MMCs are the dominant phenotype in the mucous layer, such as the gut (53). CTMCs can be found in connective tissue (e.g., intestinal submucosa, peritoneum, and skin) (1, 11, 54). CTMCs express chymase mMCP-4, trypsin (mMCP-6 and mMCP7), elastase (mMCP-5), and carboxypeptidase A3, etc. (1, 11, 54). Besides, the cytoplasm of CTMCs contains higher concentrations of heparin proteoglycan, histamine, and prostaglandin D2, while MMCs granules contain almost no heparin proteoglycan and lower levels of histamine (53, 55). Aside from the differences between the anatomic location and protease composition, MMCs and CTMCs vary in the requirement of T cells-derived cytokines, IL-3, and IL-4 (56) (**Figure 2**). Similar to mouse MCs, human MCs can be divided into two types: one subtype secretes both tryptase, chymase and carboxypeptidase A3 (MC_TC_); the other secretes tryptase alone (MC_T_) (1). The former is similar to mouse CTMCs, while the latter is equivalent to MMCs. MC_TC_ is mainly distributed in the skin and small intestinal submucosa, and participates in pathological processes, such as urticaria (57). MC_T_ is mostly expressed in the lung and small intestinal mucosa and is related to asthma and food allergy (1). In the intestine, it is reported that MC_T_ accounts for approximately 98% of the total MC population in the mucous layer but only 13% of the total MC population in the intestinal submucosa (58), while MC_TC_ accounts for approximately 77% of the MC population in the submucosa (59). Another phenotype, MC_C_, has also been mentioned in the intestinal mucosa (60). MC_C_ seems rich in chymase but deficient in tryptase (60). MC_C_ accounts for about 7% of the MCs in the intestinal mucosa and 17% in the intestinal submucosa (60); however, MC_C_ does not seem to be a unique population, given the small number of reports.

With the progression of research, it is currently considered that the existing MC classification system cannot distinguish the highly microenvironment-dependent MC heterogeneity (10, 54). For example, it has been revealed that CTMCs can express different protease phenotypes in different microenvironments within tissue locations in mice. CTMCs in trachea are mMCP-1^+^, -2^+^, -4 to 7^+^, and CPA3^+^, while those in skin and gut are only mMCP-4 to 7^+^, and CPA3^+^ (61). The ImmGen-project-derived microarray data also indicated that CTMCs subpopulations present more or less tissue-specific genetic programming differences in distinct anatomical locations, such as the skin, tongue, esophagus, trachea, and peritoneal cavity in mice (9). It has been reported that only 110 genes differentially expressed in tongue relative to trachea MCs and 122 in tongue relative to esophagus MCs. However, the difference in gene expression transcripts in peritoneal MCs relative tongue and skin MCs was 612 and 957, respectively (9). As a result, there is a growing call for a new classification method.

The plasticity of MCs in different environments has been well described. The most obvious evidence is that human MCs cultured from progenitor cells that obtained from different sites (e.g., peripheral blood, bone marrow, and cord blood) show significantly different in the expressions of receptors and granular components, and also differ in the response to the cytokines (33, 62, 63). In addition, according to the culture conditions by incubation with IL-1β, IL-4, IL-6, TGF-β1, and lipopolysaccharide in SCF-containing medium, human MCs change the protease expressions from trypsin to chymase (64, 65). In mouse MCs, co-culture with fibroblasts and stimulation with SCF induces the ability to synthesize and store heparin (66). Our previous study found that MCs co-cultured with intestinal-derived fibroblasts produce more heparin and MCPT1 and MCPT2 (10). Skin-derived fibroblasts, on the other hand, had quite different results (i.e., the increased expression of chondroitin sulfate and MCPT4 in MCs) (10, 67). According to our previous studies, there are significant differences between MCs in different organs or tissues. For example, intestinal MCs expressed high levels of the extracellular ATP receptor P2X7, which mediates the activation of MCs and worsens intestinal inflammation. In contrast, skin MCs expressed lower levels of P2X7; importantly, once the expression of P2X7 is highly induced in skin MCs, chronic inflammation occurs (10, 68). With engaging, different tissue microenvironments can produce distinct MC subtypes, which seem to be required to maintain tissue homeostasis (11). Thus, it is worth mentioning that these series of terminal maturation of MCs may receive the effect of a “peripheral immune education system” dependent on the tissue microenvironment.

The gut serves as a vast repository of agranular Lin^−^ c-Kit^+^ FcϵRIα^−^ α4β7^+^ IL-33Rα^+^ FcγRIII/II^+^ MCPs (69, 70). The gut has almost 10 times more MCPs than the bone narrow in mice (71). With the constantly-changing microenvironment, the intestinal tract is likely to create a range of diverse MC phenotypes. For example, MCs differentiate in different pathological environments and obtain a more tissue- and disease- specific phenotype relative to health conditions, which shows their extreme plasticity. Nematodes, as one of the main drivers of intestinal MCs, not only mediate the migration and expansion of MCs (71), but also induce the change of MC phenotypes. Several studies based on the *Trichinella spiralis* helminth infection model have revealed that MCs have different phenotypes at different stages of the immune response. During nematode infection, MCs in the jejunum move from the submucosa to the epithelium and sequentially expressed mMCP-2, transiently expressed mMCP-9, stopped expressing mMCP-5 to -7, and finally expressed mMCP-1 (72–74). However, in the convalescent stage of infection, MCs returned to their original site and stopped expressing mMCP-1, mMCP-2, and mMCP-5 successively, accompanied by different expression combinations of mMCP-6, -7, and -9 (72–74). The parasite repelling effect of mMCP-1 has been proven (75). In conclusion, the plasticity of MCs has been fully demonstrated by their ability to migrate at different depths in intestinal tissue for the important host defense system.

In addition to parasitic infections, increased MCs in the intestinal mucosa have also been detected in IBD (15, 76), IBS (77, 78), and food allergy (13, 79, 80). In food allergy, inoculation of food antigens leads to a dramatic increase in MMCs (due partly to infiltrating MCPs and CTMCs). Induced by TGF-β1 (81) and the Notch signaling (13) in the inflamed intestine, precursor cells mature and even undergo a reciprocal transformation of CTMCs in the submucosa and/or MMCs in the mucosa, further demonstrating the environment-dependent heterogeneity of MCs (82). Additionally, Chen et al. identified a specific group of MMCs in food allergy named MMC9 (80). MMC9 cells are a group of MMCs of Lin^−^ c-Kit^+^ FcϵRIα^+^ β_7_-Integrin^low^ ST2^+^, which can secrete large amounts of IL-9 and IL-13. This population of cells develops from MCPs that migrate to the intestinal mucosa with the help of IL-4. Under the action of IL-3 and SCF, MMC9 has the potential to rapidly develops into granular MCs and drive IgE-mediated food allergy (80, 83).

Crosstalk between MCs and surrounding cells has been widely discussed, which significantly increased the complexity of the terminal differentiation of MCs. For example, studies showed intestinal neurons could be activated by the increased histamine and trypsin secreted from MCs enriched in IBS (77) and IBD gut (76). Correspondingly, intestinal neurons mediated MCs activation by secreting neuronal factors (e.g., substance P, somatostatin, ATP, neuropeptide corticotropin-releasing factor, etc.) that stimulate MC surface receptors (e.g., MRGPRX2, P2X7, etc.) (78). The activation of MCs and their pro-inflammatory and pro-allergic mechanisms were described in more detail elsewhere (11). In addition to intestinal neurons, MCs also crosstalk with other cell types such as Tregs and intestinal epithelial cells to show the identity transformation from pathogenic or allergenic to regulatory, which plays a pivotal role in the fight against allergic responses (14) and inflammation (84). The transformation of MCs from pathogenic to regulatory roles will be discussed in more detail in the next section.

Notably, with the continuous development of technology, more comprehensive methods have been used to detect the phenotypes of different MC populations, such as single-cell RNA sequencing (scRNA-seq). In mice, a study on hematopoietic stem and progenitor cells (HSPCs) in mouse bone marrow showed that E-cadherin could be expressed by early precursors of basophils and MCs, representing the commitment to the lineages (85). Notably, scRNA-seq based on the peritoneal cavity of mice may establish an entirely new developmental trajectory for MCs, given that a discrete group of cells called P1 was found under suitable *in vitro* culture conditions, which have the potential to differentiate into MCs and basophils (86). In human, a study based on scRNA-seq revealed MCs are existed in the human yolk sac. In these human yolk sac-derived MCs, hairy and enhancer of split 1 (HES1), nuclear receptor subfamily 4 group A member 2 (NR4A2), and Kruppel-like factor 1 (KLF1) were detected, which may have non-redundant effects on the differentiation and activation of MCs (87). Another origin of human MCs was also confirmed by scRNA-seq. The analysis of human cord blood identified a group of basophil/eosinophil/MCs progenitors, revealing a close link between MCs and erythroid commitment cells downstream from HSCs (88). In addition, an analysis of nasal polyps in the patients of chronic rhinosinusitis with nasal polyposis detected an intermediate CD38^high^CD117^high^ MC phenotype distinct from circulating MCPs (12). The same study also confirmed that MRGPRX2 was expressed only in MCs of the proximal lung, but not in the distal or medial lung (12). A high ratio of tumor-suppressive TNF^+^/vascular endothelial growth factor A (VEGFA)^+^ MC phenotype is found in nasopharyngeal cancer in the patients. It seems associated with a better prognosis (89), while another ground glass nodule adenocarcinoma analysis detected pro-inflammatory MCs (90). These results clearly indicated the origin- and microenvironment-dependent heterogeneity of MCs.

Deeper analysis of innovative techniques provide sufficient resolution to fully reveal the heterogeneity of MCs and show the differentiation trajectory and transcriptional heterogeneity of specific cell types in different tissues or disease settings, which helps identify novel putative immune cell subtypes (91).

## Mast cells act as the terminator of allergies and inflammation

In pathological reactions, such as allergy and inflammation, MCs and their precursors can recruit and expand in the disease site. For example, Lin^−^ c-Kit^int/hi^ FcϵRI^+^ CD34^hi^ MCPs are recruited to peripheral tissues mediated by molecules [e.g., α4β7 integrins, VCAM-1 (92, 93)] and cells [e.g., CD11c^+^ cells (94), CD4^+^ cells (95)] in acute allergy. IgE (96) and its immune complexes (97) seem to be involved in the survival of MCs and the recruitment of MCPs. Consequently, MCs are closely involved in most allergy and inflammation processes.

Despite having outstanding achievements in innate and adaptive immunity, MCs have a well-deserved reputation as promoters of pathological actions, such as inflammation and allergy. However, as discussed previously, MCs’ distinctive migration and maturation patterns contribute to their remarkable plasticity and tissue-microenvironment-dependent heterogeneity. On this basis, we have reason to assume that MCs, as one of the most critical and primary immune cells in damaged tissues, may have positive regulatory functions in inflammation, allergy, tissue regeneration, and tissue repair. It is gratifying to note that there is now accumulating evidence to support the immunoregulatory part of MCs (**Table 1**).

**Table 1:** The immunoregulatory effect of MCs.

Mediators (Secreted by MC)	Mechanism	Reference
IL-2	Ensures the sustained and stable expression of Foxp3 in Tregs to maintain their development, proliferation, activity, and survival	(14, 98)
IL-10	Inhibits the production of pro-inflammatory and pro-allergy cytokines (TNF-α, IFN-γ, IL-1, IL-13, and IL-6)	(99, 100)
	Inhibits the expression of FcϵRI subunit protein β on MCs	(100)
	Inhibits the overactivation and over-proliferation of MCs and promotes MC apoptosis during stable and late inflammatory phases	(101–103)
	Inhibits the proliferation of T cells to inhibit inflammation	(104)
	Prevention of epithelial barrier dysfunction caused by IFN-γ and restoration of the epithelial barrier	(105)
	Inhibits adaptive immunity by suppressing the migration of mature DCs to the lymph nodes in the bladder	(106)
	Induces the production of Tregs and mediates autoantigen tolerance	(20, 107)
Amphiregulin	Boosts the Treg function in colitis and tumors in the tumor-mediated intrinsic immunosuppression vaccination model	(21)
TGF-β1	Inhibits the expression of FcϵRI subunit proteins α, β, and γ on MCs and induces MC apoptosis	(8, 17, 108)
	Induces the production of Tregs and mediates autoantigen tolerance	(20, 107)

In the early years, MCs were demonstrated to have immunosuppressive effects. For instance, Hart et al. found that in mice, dermal MCs play an indispensable role in inhibiting systemic contact hypersensitivity (CHS) response to ultraviolet radiation [wavelength 280-320 nm: (UVB)], and that histamine derived by MCs may be a critical factor for the suppression of inflammation (109). Both dermal MC-derived and exogenous histamines inhibited the immune response of UVB-exposed mice (109), through the production of PGE2. PGE2 inhibits the production of IL-12, an inducer of pathogenic Th1 immune responses (110).

In IBD, IL-33 is one of the earliest cytokines released from the necrotic cells of injured tissues (84). IL-33 has been shown to polarize AAMΦs *in vivo*, which requires the participation of MCs (22). AAMΦs have been demonstrated to have the potential to suppress inflammation and promote wound healing (111). Arginase-1 expressed by AAMΦs depletes the extracellular arginine necessary for T cell activation (112). Braune et al. and Fernando et al. found that IL-33 can induce IL-6 and IL-13 secretion by MCs through ST2, which plays a crucial role in AAMΦ polarization (111, 113). Consequently, IL-33 exerts immunosuppressive and anti-inflammatory effects by indirectly promoting AAMΦ polarization, thereby inhibiting the production of IL-17 and IFN-γ by T cells. Moreover, upon IL-33 stimulation, MCs secrete IL-10 and histamine to suppress LPS-mediated monocyte activation, which might be helpful against rheumatoid arthritis (114). Hence, it is plausible to believe that the IL-33/ST2-MC axis plays a positive role in limiting inflammation (115).

CD4^+^CD25^+^Foxp3^+^ Treg plays an essential role in immune tolerance. Several studies have shown intricate reciprocal crosstalk between MCs and Tregs. Experiments demonstrated that OX40 on the Treg surface could bind to OX40L expressed on the surface of MCs, subsequently inhibiting IgE-mediated MC degranulation (18). Moreover, TGF-β1 and IL-10 secreted by Tregs were thought to inhibit the expression of FcϵRI on the MC surface, which also inhibit the MC degranulation (17, 78, 116). Notably, TGF-β1 reduced the expression of FcϵRI subunit proteins α, β, and γ, while IL-10 inhibited only β proteins. TGF-β1 has also been reported to induce the apoptosis of MCs (108). Research on allotransplantation in experimental mice showed that Treg-derived IL-9 could maintain the tolerance of allografts by affecting MCs (19). IL-9 is a critical factor in promoting the SCF-dependent growth, proliferation, recruitment, and activation of MCs (25), thus Treg-derived IL-9 and SCF synergistically activate hepatic MCs and promote the release of histamine, IL-2, and TGF-β1 in tolerogenic liver allografts. TGF-β1 promoted the generation of γδT cells, and IL-17 released by γδT cells further attracted Tregs and enhanced their immunosuppressive properties (117).

MCs also have inverse impacts on Tregs. MCs secrete TGF-β1, which contribute to Tregs production and mediate autoantigen tolerance (20, 107). Epidermal growth factor (EGF)-like growth factor amphiregulin, which MCs also secrete, directly boosted the Treg function in colitis and tumor vaccination models by activating the EGF receptor (EGFR) on them (21). EGFR is reported to mediate intrinsic immunosuppression in tumors, and EGFR-targeted therapies are widely used for tumors such as colorectal cancer (118) and non-small cell lung cancer (119). Therefore, the immunoregulatory mechanism of MCs may be further applied in tumor therapy.

MCs also seem to ensure the sustained and stable expression of Foxp3 in Treg cells by secreting IL-2, thus maintaining the development, proliferation, activity, and survival of Tregs at sites (98) to sustain their inhibitory function (120). Studies have shown that MC-derived IL-2 in the lung promotes Tregs proliferation and limits allergic airway inflammation (115). In addition, the feasibility of regulating food allergy by inducing Treg cells with a continuous exposure of low concentration of IL-2 has been demonstrated (79).

**Figure 3:**
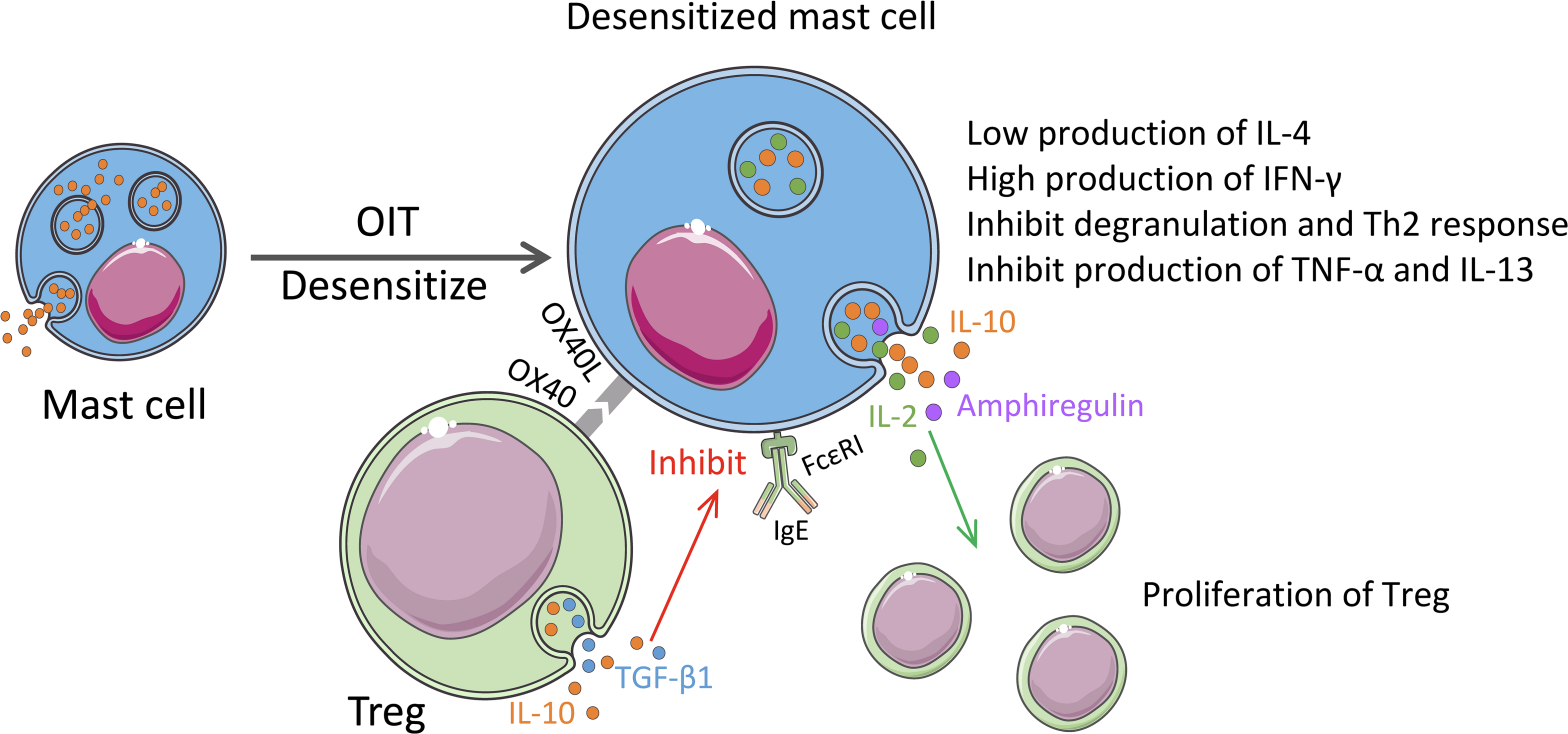
Regulatory properties of MCs in the immunotherapy. OIT treatment for food allergy induces desensitization of MCs with the low expression of Th2 cytokines and induces expression of IFN-γ. Desensitized MCs also synthesize IL-2 and IL-10. MC-derived IL-2 and amphiregulin promote proliferation and enhancement of the function of Tregs. Activated Tregs release IL-10 and TGF-β1, and the OX40 on their surface binds to OX40L from MCs, which inhibits the expression of FcϵRI on MCs and the release of TNF-α and IL-13, thereby inhibiting the Th2 responses.

Our recent research revealed the role of MCs in the regulation of food allergies (14). To delve into the mechanism of a more effective treatment for food allergy-oral allergen desensitization-oral immunotherapy (OIT) from a mucosal immune system perspective, we established a clinically relevant murine model of OIT with an escalating oral dose of ovalbumin (OVA). The OIT protocol induced mucosal desensitized MCs in the intestinal compartment with a low degranulation capacity and IL-4 production but high IFN-γ production, which plays an indispensable role in allergy (14). IL-4 plays a key role in the initiation of food allergy to mediate the isotype switching of IgE, the food-specific-IgE generation from B cells, and the release of MC mediators (e.g., histamine) (121, 122). Moreover, Tomar *et al.* found that IL-4 promotes the development of MMC9 cells, partly through a basic leucine zipper ATF-like transcription factor-dependent pathway (83). Returning to our study, we detected a significant increase in Tregs from the mucosa, peripheral blood, and spleen in OIT treated mice group. In addition, we discovered that the dual synthesis of IL-2 and IL-10 by mucosal desensitized MCs induces Tregs and inhibits allergic symptom (14). In addition, OIT treatment induce desensitization to MCs and reduced their allergenicity by acquiring the regulatory function (**Figure 3**). It can be expected that elucidation of switch pathway of MCs from allergenic to regulatory properties can lead to the utilization of MCs in a positive manner to overcome the allergy.

IL-10 is a well-known anti-inflammatory cytokine that has been demonstrated to diminish the degree or duration of innate immune or acquired responses (123) by inhibiting the production of pro-inflammatory cytokines [TNF-α, IFN-γ, IL-1, and IL-6 (99)] and chemokines, and also the proliferation of T cells [e.g., CD4^+^ T cells (123) and CD8^+^ T cells (123, 124)]. It has been reported that MCs can reduce inflammation, tissue damage, leukocyte infiltration and restore the epithelial barrier function in an IL-10-dependent manner in multiple organs or tissues [e.g., gut (15) bladder (106) and skin (16)] and the antigen-specific T cell immune response caused by *Anopheles* mosquito saliva (125). MC deficiency can increase intestinal permeability in IL10^-/-^ mice and cause spontaneous colitis (15). Artificial IL-10 supplementation prevented IFNγ-induced epithelial barrier dysfunction (105). In addition to the aforementioned Treg, MCs also secrete IL-10. Evidence of the secretion of IL-10 by MCs has been revealed both *in vivo* (14, 16) and *in vitro* (126). In Treg-independent graft-versus-host disease (GVHD), MCs have been found to show an immunosuppressive function by inhibiting T cell proliferation in an IL-10-dependent manner to inhibit inflammation (104). Although the mechanism through which MCs are triggered to produce IL-10 in the disease remains to be determined, it is speculated that endotoxin (LPS) from intestinal bacteria activates TLR4 in MCs, given that LPS has been shown to promote the production of Th2 cytokines by MCs (127). UVB promotes the production of MC-derived IL-10 by increasing the synthesis of vitamin D3 (128) to reduce allergic skin inflammation. Additionally, IL-10 derived from MCs, rather than Tregs, inhibits the migration of mature DCs to the lymph nodes in the bladder, which helps building the “immune privilege” (106).

In addition to their anti-inflammatory and allergic effects, MCs promote tissue repair and wound healing. Studies have shown that depletion of MCs partly inhibits wound healing (129). Furthermore, it has been revealed that IL-33/ST2 promoted the release of IL-13 and IL-22 from MCs (84). IL-13 signaling activates STAT6, which contributes to the survival and migration of epithelial cells and IL-22 can directly promote the production of mucus and the proliferation of epithelial cells (130, 131). Moreover, available evidence suggests that MCs are involved in almost all events of wound healing process (fibroblast migration/proliferation, and remodeling). In the early stage of wound formation, MCs are recruited to the injured site under the action of keratinocyte-derived IL-33, CCL2, and SCF; then they secrete various substances (23, 132). For example, MC produces TNF-α and stimulates DCs to express factor XIIIa, which promotes coagulation (23). Also, MC-derived histamine, lipid mediators [e.g., PGD2 and leukotrienes (LTs), etc.], and VEGF increase the vascular permeability to recruit monocytes and neutrophils (11, 23). Moreover, MCs are essential for the fibrotic process of wound healing. Proteases (e.g., tryptase and chymase), VEGF, IL-4, IL-8, NGF, FGF-2, PDGF, and TGF-β1, which are secreted by MCs, can contribute to angiogenesis, fibrin production, or re-epithelialization (23). At the end of wound healing, the proliferated blood vessels in the tissue degenerate, and the active granulation tissue is remodeled into fiber-rich scar tissue (133). Most studies agree that MCs are involved in this process (134), which might be mediated by gap-junctional intercellular communication between MCs and fibroblasts or myofibroblasts (135). In addition, MCs and zinc have been determined to induce the IL-6 production *via* Zn receptor GPR39 expressed on the cells in the inflammatory tissues such as fibroblasts, macrophages, and DCs (136). IL-6 is essential for wound healing (137).

In summary, although MCs show more inflammatory and allergic properties in the acute phase, they may exhibit more prominent functions during periods of inflammation or chronic inflammation subside to prevent excessive tissue damage and the development of chronic inflammation.

## Conclusion

In general, MCs have high plasticity and tissue microenvironment-dependent heterogeneity owing to the distinctive migration and maturation modes. MCs interact with various cells at different physiological and pathological stages to exhibit vastly different functions. Comprehensively, understanding the heterogeneity of human MCs and determining their physiological functions under specific circumstances is a great challenge. Additionally, human MCs are difficult to obtain and isolate, and have poor ability to expand *in vitro* (63). Therefore, rodent MCs are widely used for the time being, which also gives rise to a new question: whether the results observed in these alternative models can be wholly inferred to human MCs. For example, human MCs and mouse MCs sometimes show different responses to the same cytokines or anti-allergic drugs (33, 138). Clinically, due to the profound negative impression that MCs promote inflammation and allergy, until recently, simply inhibiting MCs is still the mainstream way for clinical treatment of allergy and inflammation. Moreover, it is undeniable that MC neutralizing therapies do not seem to have satisfactory therapeutic effects due to the imperfect research at the present stage. According to this review, based on the strong microenvironment-dependent plasticity and heterogeneity of MCs, it appears promising to induce MCs to behave in a manner that regulates and inhibits chronic inflammatory and allergic responses. Accumulating research evidence might have revealed the great potential of MCs in treating disease by proper education in the peripheral tissues to terminate inflammation and allergy.

## Author contributions

ZZ and YK conceived the idea for the review. ZZ and YK wrote the manuscript with constructive input from PE and HK. All authors read and approved the final version of the manuscript.

## Funding

This work was supported by grants from The Ministry of Education, Culture, Sports, Science, and Technology (MEXT) for LEADER; Japan Agency for Medical Research and Development (AMED) PRIME (20gm6010012h0004/20gm6210024h0001) and Project Focused on Developing Key Technology for Discovering and Manufacturing Drugs for Next-Generation Treatment and Diagnosis, The next-generation drug discovery and development technology on regulating intestinal microbiome (NeDDTrim) (JP21ae0121040); Japan Society for the Promotion of Science (JSPS) for Grant-in-Aid for Scientific Research S (18H05280) and Scientific Research B (19H03450), Challenging Research (Exploratory) 21K19494], Funds for the Promotion of Joint International Research (18KK0432), Future Medicine Funds at Chiba University, Danone Institute of Japan Foundation, The Naito Foundation, Hoyu Science Foundation, Waksman foundation of Japan, Yamada Science Foundation, The Institute of Medical Science, University of Tokyo (IMSUT) International Joint Research Project K003, and the Chiba University-UC San Diego Center for Mucosal Immunology, Allergy, and Vaccines (cMAV).

## Acknowledgments

We thank Mr. Izumi Tanaka for the discussion. Figures were produced using Servier Medical Art (http://smart.servier.com/), licensed under a Creative Common Attribution 3.0 Generic License (https://creativecommons.org/licenses/by/3.0/).

## Conflict of interest

HK is director and founder of HanaVax Inc.

The remaining authors declare that the research was conducted in the absence of any commercial or financial relationships that could be construed as a potential conflict of interest.

## Publisher’s note

All claims expressed in this article are solely those of the authors and do not necessarily represent those of their affiliated organizations, or those of the publisher, the editors and the reviewers. Any product that may be evaluated in this article, or claim that may be made by its manufacturer, is not guaranteed or endorsed by the publisher.
